# Constant curvature modeling of abstract shape representation

**DOI:** 10.1371/journal.pone.0254719

**Published:** 2021-08-02

**Authors:** Nicholas Baker, Philip J. Kellman

**Affiliations:** Department of Psychology, University of California Los Angeles, Los Angeles, California, United States of America; Justus Liebig Universitat Giessen, GERMANY

## Abstract

How abstract shape is perceived and represented poses crucial unsolved problems in human perception and cognition. Recent findings suggest that the visual system may encode contours as sets of connected constant curvature segments. Here we describe a model for how the visual system might recode a set of boundary points into a constant curvature representation. The model includes two free parameters that relate to the degree to which the visual system encodes shapes with high fidelity vs. the importance of simplicity in shape representations. We conducted two experiments to estimate these parameters empirically. Experiment 1 tested the limits of observers’ ability to discriminate a contour made up of two constant curvature segments from one made up of a single constant curvature segment. Experiment 2 tested observers’ ability to discriminate contours generated from cubic splines (which, mathematically, have no constant curvature segments) from constant curvature approximations of the contours, generated at various levels of precision. Results indicated a clear transition point at which discrimination becomes possible. The results were used to fix the two parameters in our model. In Experiment 3, we tested whether outputs from our parameterized model were predictive of perceptual performance in a shape recognition task. We generated shape pairs that had matched physical similarity but differed in representational similarity (i.e., the number of segments needed to describe the shapes) as assessed by our model. We found that pairs of shapes that were more representationally dissimilar were also easier to discriminate in a forced choice, same/different task. The results of these studies provide evidence for constant curvature shape representation in human visual perception and provide a testable model for how abstract shape descriptions might be encoded.

## Introduction

Shape—of contours and objects, arrangements, and environments—is fundamental to human perception, cognition, and action. An object’s shape determines its functions and often its name. A toy car or plastic horse lacks most of the crucial properties of real horses and cars, yet the categories of “car” or “horse” are nevertheless evoked by these toys’ shapes. In human perception, vision is pre-eminent in providing detailed information about shape, because it is specialized to capture spatial detail and to do so from a distance. It is not surprising, then, that visually perceived shape is central in object perception and recognition [1–4] as well as to development, learning, and concept formation [5–7]. While an inventory of sets of locations of points comprising the boundaries of objects in space would be one mathematical description of shape, research in perception has found considerable evidence that shape representations are both more and less than the local elements that comprise them [1, 8–11]. Human representations of contour shape appear to be abstractions that capture information embedded in relations, make salient similarities across objects despite differences in size and orientation, discard much irrelevant detail from stimuli presented, and require meaningful processing time to compute [12, 13].

Among the reasons for abstraction in shape representation is that objects must be recognizable across a variety of viewing conditions [14]. Research has shown that shapes and objects can readily be recognized across transformations in position [15, 16], size [17–19], and orientation, both within the picture plane [13, 20] and in depth [21]. Constancy across changes to an object’s projection onto retinae requires abstract recoding of the stimulus from a pattern of luminance contrasts at retinal positions to a symbolic, object-centric description of the spatial relationships between contour features in an object. Recent psychophysical evidence shows both the abstractness of this recoding and that it requires time for the visual system to compute beyond the processing time needed for registration of the positions of local elements [13] or the formation of a visual icon [22].

**Fig 1:**
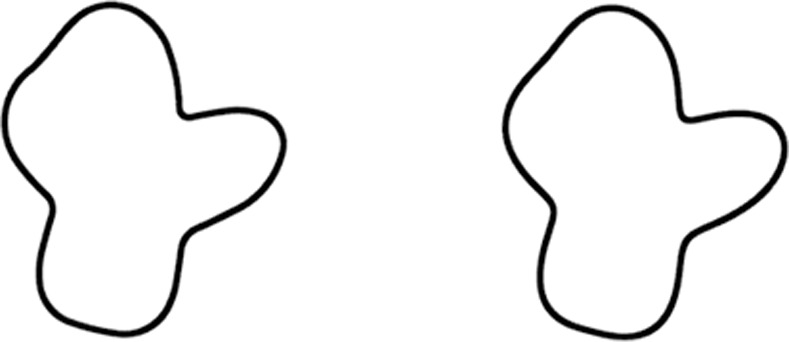
Example of shape abstraction. These two shapes differ in local curvatures at every point but are perceptually identical or very similar. The figure on the right was created by approximating contour regions in the figure on the left with constant curvature segments. Despite their differing curvature values, they have 98% shape overlap (see Experiment 3 for details) and can only be distinguished by apparent motion when a display flashes between them.

Abstraction is also important for reducing the total amount of information along an object’s contour. Compared to a much larger amount of raw information available immediately after stimulus registration [23, 24], a much smaller amount is encoded in more durable memory stores [25]. Experiments testing detection of contour differences suggests that some contour features are not encoded in our shape representation (e.g., [26]). As another example, consider Fig 1. Although the two shapes have none of the same curvature values, they are visually indistinguishable (or nearly so). Attneave proposed that the visual system preferentially encodes regions of high curvature along a contour, arguing that, from an information theory perspective, high curvature areas are more informative about the object’s shape [27, 28].

Many models of shape representation have been proposed, both from work in human perception and computer vision. For two-dimensional (2D) shapes, one prominent theory is that shapes are encoded as a set of skeletal branches that capture areas of local symmetry along a contour. This idea originated with Blum, who proposed the medial axis transform (MAT), which recodes a set of contour points to a set of axial branches [29]. The MAT is completely data-driven and does not abstract over any perturbations along a contour, no matter how small or imperceptible. It can therefore capture potentially important perceptual features of a contour, but it does not simplify the contour representation, nor would it be robust to small, imperceptible changes to an object’s contour. Newer skeletal models have corrected this, putting contour reconstruction accuracy in tension with representational simplicity [30]. These models are theoretically rigorous, but experimental evidence relating them to human perception has been limited. A few recent studies have found evidence that participants’ similarity judgments for objects correlate with the objects’ skeletal similarity [31–34]. Crucially, however, the stimuli used in these experiments were generated from skeletal branches. For shapes not explicitly generated from skeletons, some research has found that Bayesian shape skeletons can capture differences from superordinate categories with relatively few parameters [35] and help pick out important features that are most relevant to categorization of a natural scene [36]. When testing with novel 2D contours, a different study found no difference in participants’ performance in a same/different task for two shapes that differed in the number of branches given by skeletal axis transformation (a qualitative difference) vs. shapes that had the same number of branches but differed only in some change of curvature along a branch (a metric difference) [37]. These findings may suggest that skeletal shape representations contain important information for high-level object and scene perception but might not capture contour differences perceptually relevant to mid-level contour encoding.

Other contour-based models of 2D shape representation have also been put forward. Hoffman & Richards proposed that deep concavities along a contour determine how a shape is decomposed into parts [38]. Subsequent research has found support for the notion that curvature minima are more salient than curvature maxima along a contour [26]. Kass, Witkin, and Terzopolous modeled shape representations as a series of deformations of a basic shape primitive [39]. They used evolving splines to capture these deformations. Several newer models have used the same idea of template deformation but adjusted the algorithms to make the shape representations more robust to local contour changes and partial occlusion [40, 41]. These impressive models, originating from computer vision, use sophisticated mathematical tools to capture shape representation. It is not clear how relevant these models are to biologically plausible theories of shape representation.

Another model of shape that has been used in computer vision research involves dividing a contour into a set of line segments, then merging together adjacent segments that are sufficiently similar in orientation [42]. Gdalyahu and Weinshall showed how such *split-and-merge* techniques with line segment primitives could be used as the basis for object recognition algorithms in natural images [43]. Similar split-and-merge methods have been proposed in computer vision with splines rather than straight segments to more efficiently code curvature along a contour [44].

## Constant curvature representations of shape

A particularly interesting possibility that we have pursued in recent research is that contour shape may be represented as sets of contour segments of constant curvature [45, 46]. This idea had been proposed earlier in computational vision by Wuescher and Boyer, who developed an algorithm for constant curvature segmentation of contours and showed that they fit contours better and with fewer primitives than straight segment approximations [47].

In biological vision, the idea that 2D shape representations are made up of smoothly joined constant curvature segments is intriguing for several reasons. One is that this idea may be consistent from some evidence obtained in single unit recording in the primate visual brain. Pasupathy and Connor found evidence of neuron populations in V4 that are sensitive to specific curvatures that might be primitives of more complex shape representations [48, 49]. From an ecological perspective, a great deal of work in natural scene statistics has examined the prevalence of co-circular contours in our visual environment [50, 51]. While results differ on how many truly co-circular contours exist in the visual environment, there is agreement on the prevalence of curved, nearly co-circular contours that could be well-estimated by constant curvature encoding.

Garrigan & Kellman found psychophysical evidence for the use of constant curvature primitives in contour representation [45]. Open contour fragments made up of constant curvature segments were encoded more accurately at brief viewing durations than fragments made up of non-constant curvature. They attributed this to the greater similarity between the physical properties of the constant curvature contour and participants’ abstract representation of the contour. More recently, Baker, Garrigan & Kellman tested the hypothesis of constant curvature primitives in contour representation in several different experimental paradigms, showing that constant curvature paths are easier to detect in visual search than non-constant curvature paths, and that people can learn to segment a contour made of two constant curvature segments much more accurately than a contour made of two segments with different physical properties. They also described how constant curvature segments might be obtained from neural units, known to exist in early visual cortical areas, that code orientation at multiple scales [46].

These results implicate constant curvature as having a special role in shape representation. A more complete explanation of the role of constant curvature would involve a model of how such representations are obtained from visual stimuli. In the current work, we developed a computational model for how this could be accomplished, tested its plausibility, estimated its parameters from psychophysical data, and then further tested the fully specified model.

### Modeling of constant curvature shape

Garrigan proposed a computational model for how a 2D contour might be represented by a set of smoothly joined constant curvature segments [52]. We briefly review the model here as a basis for the experimental efforts and model specification put forth here. The constant curvature model takes an object’s bounding 2D contour as an input and outputs a representation of the shape made up of a small number of constant curvature primitives.

The model begins by computing the signed curvature at every point along the inputted shape contour. For a plane curve defined in parametric form by the equations x = x(t), y = y(t), curvature k = || dT/ds || is calculated as $\frac{\left|{\text{x}}^{\prime }{\text{y}}^{\prime \prime }-{\text{y}}^{\prime }{\text{x}}^{\prime \prime }\right|}{{\left[x^{\prime }{)}^{2}{\left(y^{\prime }\right)}^{2}\right]}^{3/2}}$, where t specifies a point along the curve, || dT/ds || is the norm of the change in the unit tangent vector T per unit arc length s, x is the horizontal component of the curve and y is the vertical component of the curve, and x′ and y′ and x″ and y″ denote the first and second derivative of the x and y components respectively.

Next, the contour is segmented into regions of similar curvature by identifying points at which the curvature changes from higher than the local average to lower than the local average, or vice versa. The segmentation process considers all adjacent points, *a* and *b*, along a contour and places a segmentation boundary between them if the difference between the curvature at *a* and the mean curvature in a local window centered on *a* is positive and the curvature at *b* and the mean curvature in a local window centered on *b* is negative, or vice versa. The precision of this segmentation depends on the size of the local window, which we term the integration window, *W*, with which the curvatures at *a* and *b* are compared. Formally, the model will add a segment boundary between a and b if:

$$\left({\text{k}}_{\text{a}}-\frac{1}{2W+1}\sum_{i=a-W}^{i=a+W}k_{\text{i}}\right)*\left({\text{k}}_{\text{b}}-\frac{1}{2W+1}\sum_{i=b-W}^{i=b+W}k_{\text{i}}\right)<0$$ (1)

Here, *W* represents the amount of contour considered when deciding if a segment boundary exists between *a* and *b*. Mean curvatures are calculated in the interval (*a*–*W*, a + *W*) and along the interval (*b*–*W*, b + *W*). Larger values of *W* correspond to larger windows that are therefore more tolerant to variance in curvature when the model decides whether to partition the contour between points *a* and *b*. Smaller values correspond a smaller window that is less tolerant to curvature variation.

Once the segment boundaries have been identified, the model recodes all contour points between the segment boundaries into a single constant curvature segment. A constant curvature segment is a contour region in which all points within the region are represented with the same curvature. It is described by an object-centric spatial position, a signed curvature (defined as the mean of all curvatures between the segment boundaries), and an arclength.

Curvatures of adjacent segments are then compared to ensure that all segments are sufficiently dissimilar to merit a separate primitive representation. If the difference in curvature between any adjoined segments is below a threshold value, *T*, then the visual system will represent the pair of segments as a single primitive. The curvature of the merged segment will be the mean of the curvature of the two segments, weighted by their respective arclengths. This process continues until no pair of adjacent segments have a curvature difference below *T*. In mathematical terms, if

$$\left|k_{2}-k_{1}\right|<T$$ (2)

where *k*_*1*_ and *k*_*2*_ are the curvatures of adjacent segments, then the visual system will merge them into a single constant curvature segment with the weighted mean of their curvatures, given by

$$k_{12}=\frac{l_{1}*k_{1}+l_{2}*k_{2}}{l_{1}+l_{2}}$$ (3)

where *l*_1_ and *l*_*2*_ are the lengths of the adjacent segments. Here, *T* specifies the visual system’s sensitivity to differences in curvature between constant curvature segments.

The final representation is the set of constant curvature segments composing the shape, each described by a position, curvature, and arclength (see [52] for a more detailed description of these aspects of the model).

The constant curvature model includes two free parameters: the size of the integration window (*W*) used in segmentation and the minimum difference in curvature (*T*) needed for two adjacent segments to be represented separately. Both of these parameters balance the representation’s fidelity to the original contour with a preference for simpler representations built up from fewer primitives. By fidelity, we refer to the physical difference in positions of points along the contour for a constant curvature representation of the contour compared to the physically given contour. By simplicity, we refer to the number of constant curvature segments from which the constant curvature representation is formed. This tension is a classic concern in research on visual perception [53–55]. Research on *minimum tendencies* has found that the visual system tends to represent visual information as simply as possible, up to a certain loss in fidelity to the original stimulus [56–58].

In shape perception, this tension has been formulated in Bayesian terms as the balance between a simplicity prior, where, in the absence of data, a representation that is more complex has less *a priori* probability, and a likelihood, where a representation is more probable if it more closely matches the original contour [30]. The best representation, then, is one that matches the original contour reasonably well, but does not represent contour features that greatly increase the representational complexity while only marginally improving the representational fidelity. Note that the prior specified in Feldman and Singh’s model has no relationship with frequency statistics in typical visual environments. Their Bayesian formulation is mathematically equivalent to non-probabilistic models that define cost functions for both complexity and differences from the true contour. Of course, a Bayesian framework still has flexibility in how the prior and likelihood are quantified. A model with a narrow prior distribution and a wide likelihood distribution will emphasize simplicity, while one with a wide prior distribution and a narrow likelihood distribution will emphasize fidelity.

In the constant curvature model, fidelity and simplicity are balanced by the values fixed to the integration window size (*W*) and curvature difference (*T*) parameters. For *W*, using a smaller window results in more segment boundaries approximating the original contour. Representations formed from small window sizes therefore have higher fidelity but more complexity than representations formed from larger window sizes. For *T*, a larger threshold results in the combination of segments with larger curvature differences, resulting in fewer primitives but greater difference between the physical and represented curvature in a region of the contour.

In order to test specific predictions of the constant curvature model, both of its free parameters must be specified. It is an open question how consistent the parameters are across people and viewing conditions. Garrigan hypothesized that they are flexible, and that the visual system uses smaller parameters for visual tasks that require a high degree of specificity, and larger parameters for tasks in which a loose approximation of the shape is adequate [52]. On the other hand, if parameters are truly believed to vary flexibly, the visual system would be unable to match same shapes that were viewed under different task conditions. It is possible that additional perceptual resources could be allocated for tasks that require an extremely high degree of shape fidelity, but it seems likely that the visual system always encodes a representation with a fixed level of specificity for general recognition.

In the current work, we tried to estimate and evaluate the constant curvature model specifically as used for shape recognition. In Experiments 1 and 2, we used psychophysical experiments with simple open contour stimuli to fix the curvature difference threshold (Experiment 1) and integration window size (Experiment 2) in the computational model for constant curvature representation. In Experiment 3, we used the parameters fixed in Experiments 1 and 2 to test the empirical validity of the fully parameterized constant curvature model.

## Experiment 1

In Experiment 1, we aimed to specify the threshold parameter (*T*) of the constant curvature model. The threshold parameter determines the point at which two adjacent constant curvature segments are represented as a single segment based on the difference in curvature between them. In other words, if the curvature difference between two smoothly connected segments is less than the threshold parameter, then the visual system should encode them as a single segment of constant curvature that extends the length of both constituent segments. The parameter acts in service of the simplicity constraint for the constant curvature model, ensuring that a shape is represented by as few primitives as possible provided that the representation still has sufficient descriptive capability to support recognition, discrimination, motor action, and reasoning about functional properties.

We devised a simple psychophysical experiment to measure the visual system’s sensitivity to differences in curvature between two smoothly joined constant curvature fragments. Previous work by Baker et al. found that the visual system is capable of accurately separating two constant curvature segments at their point of transition, but not two segments of constantly accelerating curvature [46]. In this experiment, we varied the difference in curvature between two constant curvature (CC) segments in order to determine the curvature differences that lead to two segments being perceived as a single curvature as opposed to a composition of two distinct curvatures. We hypothesized that the maximum curvature difference that is undetectable to participants would be a natural threshold for the constant curvature model in deciding whether two adjacent segments should be represented as one or two segments of constant curvature.

## Method

### Participants

Research on human participants’ was approved by the UCLA Institutional Review Board IRB#11-002079-CR-00001. Participants gave oral consent before beginning the experiment. Twenty-six undergraduates (eight male, 18 female, *M*_age_ = 20.6) from the University of California, Los Angeles participated in Experiment 1 for course credit. All participants had normal or corrected-to-normal vision.

### Stimuli

In each trial, we generated two open contour stimuli, one made up of a single constant curvature segment, and one made up of two smoothly joined constant curvature segments. The single CC contour was generated with a random length and curvature. The length of the contour was between 240 and 500 pixels (5.76–12 degrees of visual angle), and the curvature was between 0.0059 and 0.02 pixels^-1^ (see [59] for more information on units of curvature), which corresponded, at the viewing distance used, to curvatures between .004 and .0134 arcmin^-1^. The mean length of contours for both conditions was equated. The angular extent of the single CC contour was determined by its length and curvature, but we added a constraint that the angular extent must be less than 360 degrees.

**Fig 2:**
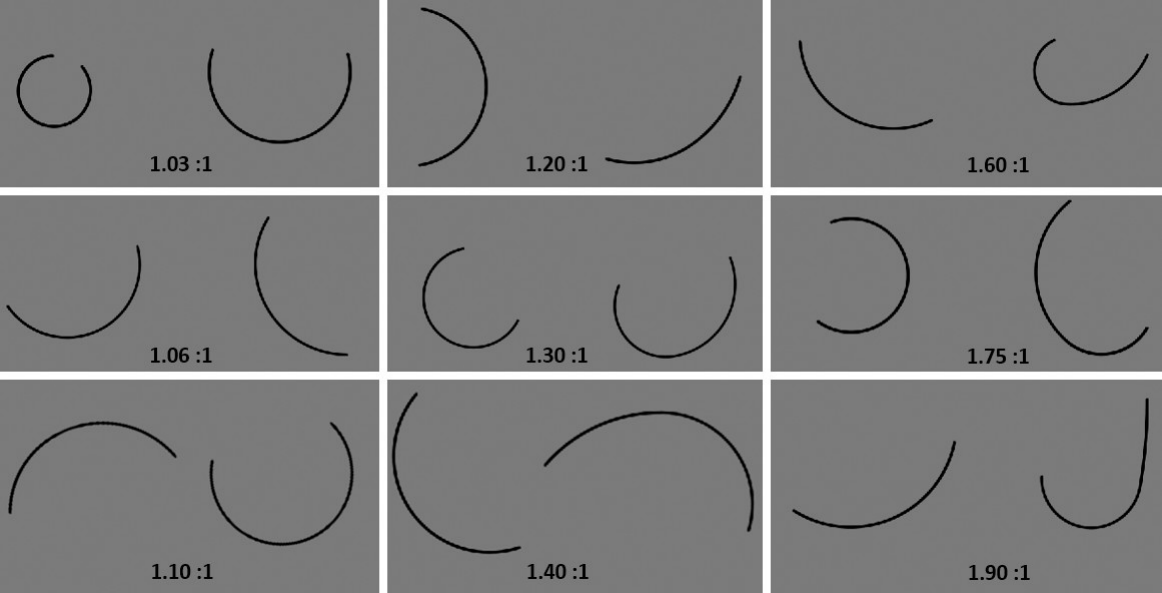
Sample trials from Experiment 1. Participants were shown two open contours side-by-side and asked which was more complex. In each display, one contour had a single curvature while the other had two curvatures. The different curvature ratios are shown in the nine panels. For display purposes here, the two contour fragments are presented closer together than in the experiment, the two CC contour is shown to the right of the single CC contour, and the ratio is shown in each panel.

The contour made of two connected CC segments was created by generating one constant curvature segment with random length (between 120 and 250 pixels) and curvature (between 0.0059 and 0.020 pixels^-1^), then smoothly connecting a second segment to it. For the contour to be differentiable at all points, the second segment had to begin at the same angular position at which the first segment terminated. The second segment also had random length between 120 and 250 pixels. The curvature of the second segment was determined by the first. We varied the difference in curvature across nine conditions as a ratio. The possible curvature ratios ranged from 1.03:1 to 1.9:1. The order of the two segments was randomized so that half the time the higher curvature segment was clockwise of the lower curvature segment, and the other half it was counterclockwise. Fig 2 shows sample trials of Experiment 1.

### Display and apparatus

Participants were seated 70 cm from a 20-in. View Sonic Graphic Series G225f monitor. The monitor was set to 1024 x 768 resolution, with a refresh rate of 100 Hz. All stimuli were black contours shown on a gray background. One contour was shown in the center of the left half of the screen, and the other was shown in the center of the right half of the screen.

### Design

Experiment 1 had 225 trials, consisting of nine conditions with 25 trials each. The nine conditions corresponded to the ratio of curvatures between the two constant curvature segments from the two CC open contour. The nine ratios were 1.03:1, 1.06:1, 1.1:1, 1.3:1, 1.4:1, 1.6:1, 1.75:1, and 1.9:1. The conditions were ordered in blocks from the highest ratio to the lowest so that better performance for higher ratios could not be explained by practice effects. Participants completed five practice trials with the experimenter present to ensure that they understood the instructions before beginning the main experiment.

### Procedure

In each trial, one open contour with a single curvature segment and one open contour made of two curvature segments were shown simultaneously, one in the left half of the screen and one in the right half. Position was randomly assigned in each trial. Participants were told to look at both open contours and judge which one they believed was more complex. They were then told to press “A” if they believed the contour on the left was more complex, or “L” if they believed the contour on the right was more complex. No explanation was given about what was meant by complexity, but the more complex stimulus (i.e., the open contour made of two constant curvature segments) was highlighted in blue in each trial after participants had given their response. Participants were free to look at the stimulus pair for as long as they wanted before responding.

## Results

**Fig 3:**
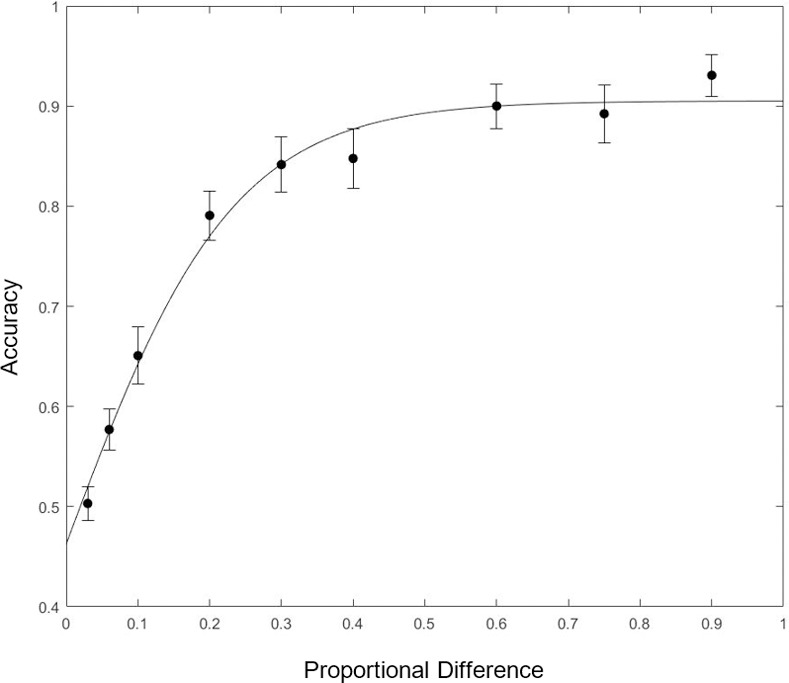
Performance as a function of curvature difference. The horizontal axis gives the proportional difference between curvatures for the two-segment contour fragment. For example, if the proportional difference is 0.1, then the curvature ratio between the two segments is 1.1:1. Error bars indicate ±one standard error of the mean.

Mean accuracy results are plotted for each curvature difference in Fig 3. Performance was at chance for a ratio of 1.03:1 (*t*(25) = 0.18, *p* = .83, 95% CI = [.47, .54]). At a ratio of 1.06:1, performance was marginally better than chance after correcting for multiple comparisons (*t*(25) = 3.71, *p* = .01, 95% CI for performance = [.53, .62]). For all other curvature ratios, participants performed reliably better than chance even with a Bonferroni correction for multiple comparisons.

Performance improved rapidly with larger curvature ratios up to a ratio of 1.3:1, after which it flattened out and improved only marginally as the ratio got larger. This suggests that the critical point at which the visual system encodes two contours of similar curvature as different occurs somewhere between a ratio of 1.03:1 and 1.3:1. Since chance was 50%, we found the 75% performance threshold to estimate the value of *T*. We fit a psychometric function to the data using the Palamedes Toolbox [60] and found the 75% threshold to be a curvature ratio of 1.18:1.

**Fig 4:**
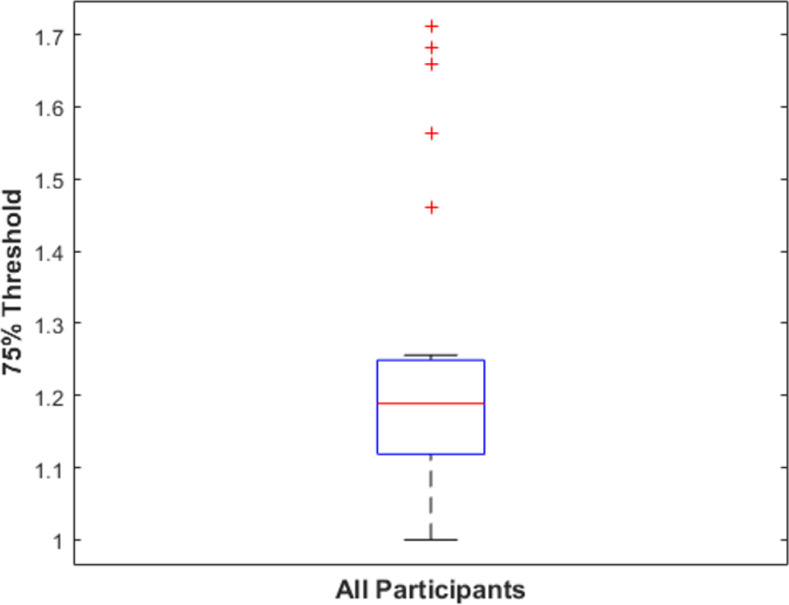
Boxplot of participants’ 75% threshold in Experiment 1. The box shows the interquartile range of thresholds for individual participants. The red line shows the sample median. The whiskers extend to the most extreme datapoint within 1.5 times the length of the interquartile range from the top or bottom edge of the box (covering 99.3% of the data if they are normally distributed) [61, 62]. Outliers are data points beyond the whisker and are plotted as red +’s.

We also assessed the consistency of results across participants. We fit a psychometric function to individual participants’ data using the same method as used for the group data to determine each participant’s individual 75% performance threshold. In Fig 4, we show a boxplot of individual participants’ 75% threshold. We found that individual participants’ thresholds were generally very similar to the threshold obtained from the group data. The average threshold for individual participants was 1.24, and the standard deviation of the mean was 0.20. Of the 26 participants, 12 had a threshold within 0.05 of curvature ratio obtained from the group mean, and 19 of 26 had a threshold within 0.1 of the group mean.

## Discussion

A classic approach to characterizing sensory systems in psychophysics involves identifying the smallest detectable physical difference between two stimuli [63, 64]. In Experiment 1, we used an implicit version of a just noticeable difference procedure to evaluate participants’ perceptual ability to detect differences in curvature. Unlike much traditional work in psychophysics, this study did not test participants’ sensitivity to differences in sensory magnitudes (e.g., brightness or loudness), but to object features.

Our results showed a systematic relationship between limits in resolving differences in contour segments and curvature ratios. Participants did not reliably perceive the contour made of two constant curvature segments as more complex when the ratio was 1.03:1. Contours with a curvature ratio of their parts of 1.06:1 were judged more complex than constant curvature stimuli only marginally better than chance, presumably because these stimuli were often encoded as a single segment of constant curvature. On this account, as the ratio between curvatures increased, the probability of participants encoding the contour as having two curvature values increased, resulting in more accurate selection of one contour as more complex. Participants attained near-ceiling accuracy when the curvature ratio was about 1.3:1.

An important design feature of this experiment was that participants were instructed to choose the more complex stimulus, not the stimulus with more than one curvature. In fact, no mention of curvature was made at any point during Experiment 1. This allowed us to test the role of curvature differences without suggesting particular strategies to participants, and it also made it possible to see spontaneous effects of curvature differences on perceptual judgments of complexity. Still, for all but the most similar curvature pairs, participants were consistent in choosing the contour made of two curvature segments as the more complex stimulus. Participants did receive feedback after each trial, which they may have used to infer what we meant by complexity, but it would still need to be perceptually salient for participants to respond as accurately as they did. Moreover, the condition in which participants had the highest average accuracy was completed in the first block of trials, suggesting little learning was needed to judge complexity in terms of number of constant curvature segments.

The ease with which participants used curvature difference as an indicator of stimulus complexity furnishes additional evidence, beyond earlier work (e.g., [45, 46]) that constant curvature segments are indeed basic units of contour shape representations. If a shape is represented by a set of primitives, it stands to reason that shapes built up from more primitives are more perceptually complex than shapes built up from fewer primitives.

Other notions of contour complexity would not make the same prediction. Hoffman and Richard hypothesized that a shape is decomposed into parts based on the presence of curvature minima [38]. By this definition, the contours in Experiment 1 all had the same number of parts, as the sign of curvature never changed for either stimulus category. Our data suggest that there are perceivable differences in shape complexity even when part numbers are the same. Another account of contour complexity posits that stimuli with higher curvature are more complex [27, 28]. In our study, the single-segment contour had higher curvature than the two-segment contour 50% of the time, but participants reliably chose the two-segment contour as more complex. One explanation for this is that the visual system does not have lowest surprisal when a contour continues straight in the tangent direction as has been suggested [28], but when its curvature is most similar to the curvature of contour areas nearby it. Another possibility is that viewers have different notions of contour complexity that they can flexibly choose between when comparing contours. Because we gave participants feedback after each trial, they may have learned to use a notion of complexity that depends on the amount of curvature variation in a contour fragment, but with different feedback they could have learned to judge complexity based on deviation from a straight continuation of the contour or number of local minima. Which notion of complexity viewers default to without feedback is an open question, but the results of Experiment 1 suggest that a notion based on curvature variation is available to, and easily used by, the visual system.

The results of Experiment 1 help us to fix the threshold parameter for the constant curvature model. The segment merging operation in our model is deployed after an initial segmentation of the contour into constant curvature segments has already been completed. It serves to prune the shape representation by encoding adjacent segments of similar curvature with a single CC segment. Two segments are merged into a single primitive if the difference in curvature between them is below a certain threshold. A likely candidate for what this threshold might be is the point at which two curvatures are detectably different more often than not. By fitting a psychometric function to the Experiment 1 data, we found the 75% threshold as an estimate of when curvature differences are reliably detected. This point corresponds to a curvature ratio of 1.18:1. The high degree of consistency among individual participants in Experiment 1 suggests that this ratio is common among all observers, not just the mean point between observers with a diversity of individual thresholds.

In Experiment 1, we used only segments of constant curvature. In the world, object contours have far more curvature variation [51]. For our purposes, however, restricting our stimuli to one or two segments of constant curvature is more directly relevant to how the threshold parameter works in the constant curvature model. The threshold parameter merges segments of similar curvature after they have already been segmented and recoded into constant curvature primitives (see Model), so we restricted our stimuli to contours that were plausible inputs at that stage of the constant curvature model.

## Experiment 2

In Experiment 2, we carried out an experiment to estimate the second free parameter of the constant curvature model: the size of the integration window used in segmenting a contour into constant curvature regions. In our model, the integration window size is parameterized as *W*, and corresponds to a percentage of the contour’s total length. *W* determines how contour regions with non-constant curvature are approximated by a relatively small set of constant curvature segments. In the constant curvature model, the visual system assigns adjacent points along a contour, *a* and *b*, to different segments if the difference between the curvature at *a* and the mean of curvature within the integration window centered on *a* is positive and the difference between *b* and the mean of curvature within a window centered on *b* is negative, or vice versa (Eq 1). *W*, then, determines how finely or coarsely a contour is segmented into regions of constant curvature.

**Fig 5:**
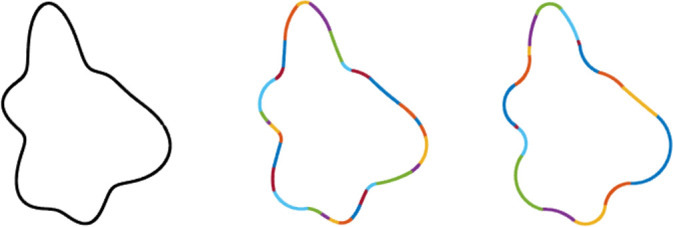
Two constant curvature representations of a shape contour. The original shape (left) is approximated with a small *W* (middle) and a large *W* (right). The smaller integration window has more segments and is more precise, organizing regions of the original shape with less variance in curvature into a single constant curvature segment (mean standard deviation of curvature = 0.02 per segment), while the larger integration window has fewer segments, but represents the contour less precisely, organizing regions with more curvature variance into single constant curvature segments (mean standard deviation of curvature = .05 per segment).

If the model uses large integration windows, a higher percentage of contour points will be shared in the integration window centered on *a* and the integration window centered on *b*, so a contour boundary will be drawn more rarely between two points. Consequently, model outputs with large *W* will tend to be coarser, abstracting over more curvature variety along an object’s contour. On the other hand, models in which *W* is small yield outputs with more constant curvature segments, which are consequently much more visually similar to the original object boundary (see Fig 5).

**Fig 6:**
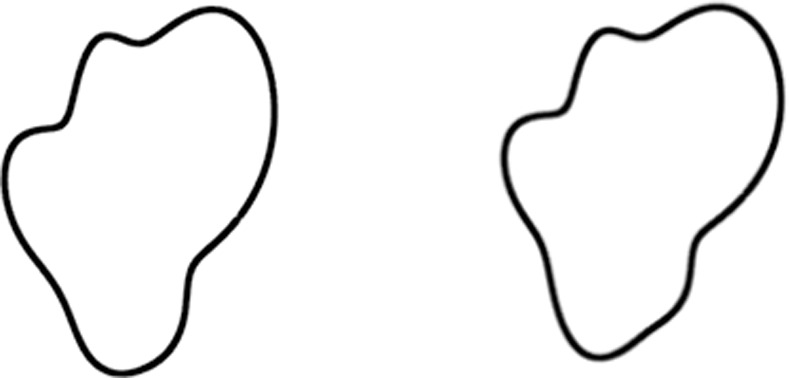
Shape pair with similar contour features. The first member of the pair was generated by moving 12 control points in a circle towards or away from the center and fitting cubic splines between the 12 control points. The second member of the pair was generated by moving two adjacent control points from the first member a random direction and fitting a new set of cubic splines between the resulting dozen control points.

In the extreme, the visual system would represent every unique curvature along an object’s boundary with its own CC segment. This would give a perfect reproduction of the contour but would also likely tax the visual system far beyond the capacities of visual memory [65]. More likely, the visual system abstracts over some curvature variation, but encodes a precise enough representation to allow it to discriminate between similar but nonidentical shapes, such as those in Fig 6.

The degree of precision with which contours are encoded is an empirical question. If the visual system is segmenting contours into constant curvature segments and encoding the constant curvature representation, there should be a point at which the constant curvature representation of a contour is indistinguishable from the original contour in a visual memory task. To determine this point, we compared participants’ ability to discriminate constant curvature representations of contour fragments from the real fragment across a variety of integration window sizes. We hypothesized that the visual system uses the largest integration window for which the model output is indistinguishable from the inputted contour, since that will be the window size that is most economical while also representing the shape with sufficient precision.

## Method

### Participants

Twenty-three undergraduates (7 male, 16 female, *M*_age_ = 20.6) from the University of California, Los Angeles participated in Experiment 2 for course credit. All participants had normal or corrected-to-normal vision.

### Display and apparatus

All display conditions were the same as in Experiment 1.

### Design

Experiment 2 consisted of nine conditions, corresponding to nine integration window sizes. We initially specified window size as a contour length in degrees of visual angle. The nine sizes were 0.32, 0.64, 0.97, 1.29, 1.61, 1.93, 2.25, 2.58, and 2.90. There were 40 trials for each condition. On the trials of most interest, an open contour that was not made of constant curvature parts was shown initially, and participants then had to make a forced choice between two contours, one of which was the initially presented contour and one of which was a representation of that contour made from constant curvature parts. In addition to these 180 experimental trials, we also had 180 trials (20 using each integration window size) in which the initially presented contour fragment was made of constant curvature (see Procedure). Results from these trials were not included in our primary analysis. Before beginning the experiment, participants completed 10 practice trials.

### Stimuli

Each trial included a contour fragment with nonconstant curvature. The contour was obtained by first generating a closed contour by displacing 12 control points along a circle and fitting cubic splines between the control points (see Fig 6 for two examples), then taking a fragment from the closed contour, totaling 40% of the closed shape’s overall contour length, on average 12.88 degrees of visual angle. Every trial also had a constant curvature representation of the contour, generated with the fixed threshold parameter from Experiment 1 and various integration window sizes specified by the nine trial conditions.

### Procedure

**Fig 7:**
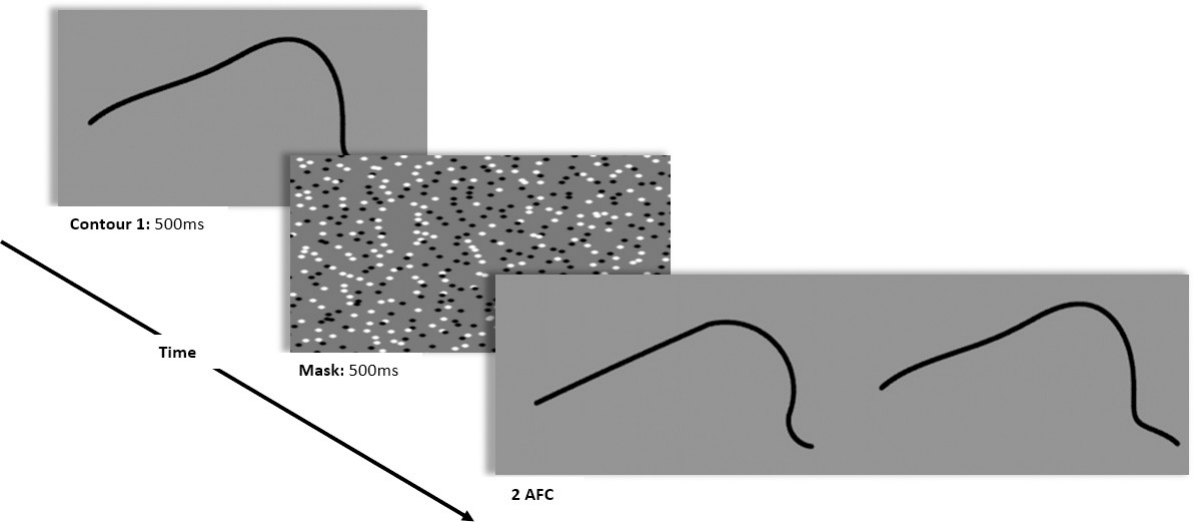
Sample trial for Experiment 2. The nonconstant curvature contour was shown first, followed by a mask. Then, a constant curvature representation and the original contour were shown side-by-side. Here, the constant curvature representation is generated with a window size of 2.25 degrees of visual angle.

In the analyzed trials, participants were shown a fixation cross in the center of the screen for 300 ms, followed by the nonconstant curvature contour fragment for 500 ms. A pattern mask was shown for 500 ms after exposure to the contour fragment, after which participants were shown two contours simultaneously (one in the center of the left half the screen, one in the center of the right half of the screen) and asked which one exactly matched the first contour they had been shown. One of the two contours shown after masking was identical to the first contour. The other was the constant curvature representation of the contour generated with an integration window size determined by the trial condition (see Fig 7 for a sample trial). In the analyzed trials, the correct response was always the nonconstant curvature contour fragment. participants could view the two contours for as long as they wished before responding.

To prevent participants from using a strategy in which they always pick the contour fragment with constant curvature without comparison to the original contour, we also included 180 trials in which the constant curvature representation of the contour fragment was shown first instead of the nonconstant curvature contour fragment. After masking, the constant curvature contour and the nonconstant curvature contour were shown as in the main trials, but the correct response for the matching shape was the constant curvature contour.

## Results

**Fig 8:**
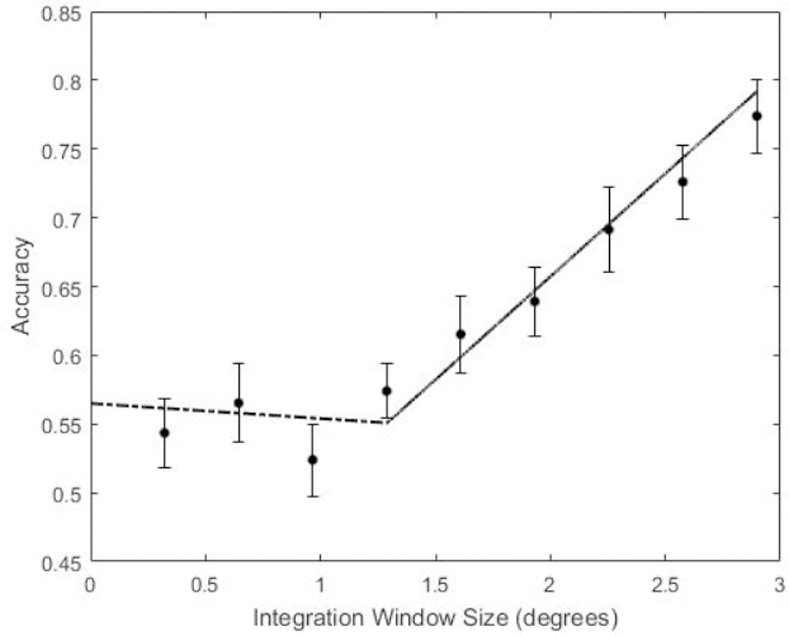
Results from Experiment 2 with best-fitting piecewise linear regression. The dashed line shows the linear fit up to the transition point and the solid line shows the linear fit past the transition point. Error bars indicate ±one standard error of the mean.

The results of Experiment 2 are shown in Fig 8. After correcting for multiple comparisons, participants were at chance performance for the three smallest integration window sizes (*t*(21) = 1.75, *p* = .09, 95% CI = [.49, .59], *t*(21) = 2.25, *p* = .03, 95% CI = [.51, .63], *t*(21) = .91, *p* = .37, 95% CI = [.47, .57], respectively). Starting at an integration window size of 1.29 degrees, participants were able to reliably distinguish the constant curvature representation from the original contour fragment (*t*(21) = 3.77, *p* = .001, 95% CI = [.53, .61]). Performance improved monotonically for larger integration window sizes.

Unlike the data from Experiment 1, which followed an S-shaped function, the data in Experiment 2 appear to be well described by two linear functions, one approximately flat for integration windows that are all indistinguishable from chance guessing, and another function with positive slope beginning at the point where the constant curvature representation became coarser than the shape representation encoded in visual memory. Because we were looking for the very first point in which accuracy as a function of *W* is described by a positive slope, we analyzed the data for a change in slope polarity rather than looking for the 75% threshold as we did in Experiment 1. We looked for the largest integration window size at which participants begin to detect that the constant curvature representation of the shape was different than the original shape. This corresponds to a transition point in a continuous piecewise linear regression model from zero (or, in our case, slightly negative) slope to a positive slope. To identify this transition point, we fit the data with several continuous piecewise linear regression models, specifying different sizes of *W* at which the slope changes in order to determine which one explained the most variance. *R*^*2*^ was highest (.299) when the slope changed at *W =* 1.29 degrees of contour length, (*F*(2, 204) = 43.52, *p* < .001). For this regression, the slope before the transition point is not significantly different from zero (*t*(2) = -0.69, *p* = .49), while the slope beyond the transition point does differ significantly from zero (*t*(2) = 2.69, *p* = .008).

**Fig 9:**
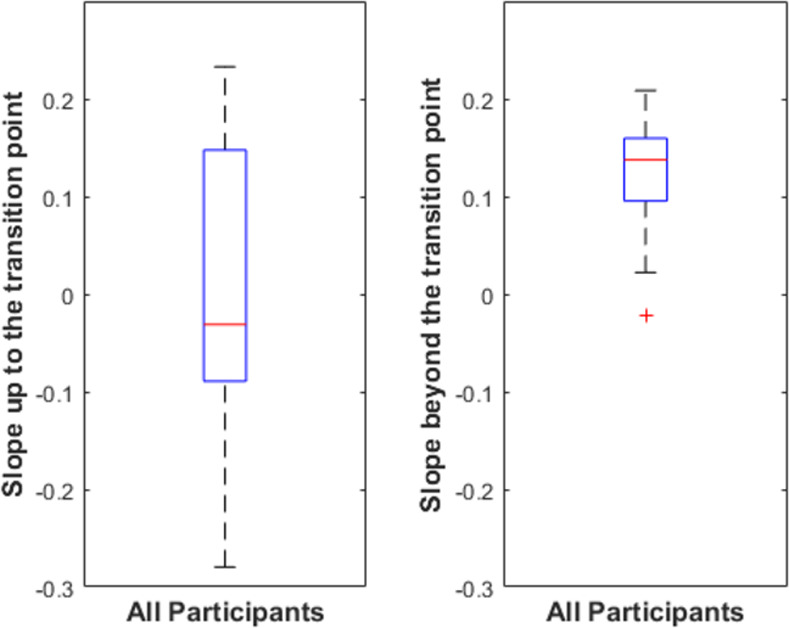
Boxplot of participants’ performance slopes up to and beyond the transition point. The box shows the interquartile range of slopes for individual participants. The red line shows the sample median. The red line shows the sample median. The whiskers extend to the most extreme datapoint within 1.5 times the length of the interquartile range from the top or bottom edge of the box (covering 99.3% of the data if they are normally distributed). Outliers are data points beyond the whisker and are plotted as red +’s.

We performed additional analyses of the Experiment 2 data to assess consistency among individual participants in their ability to distinguish constant curvature representations from original contours at different degrees of representational fidelity. We did this by computing the slope of participants’ performance on either side of the transition point (*W* = 1.29) and testing whether the slope beyond the transition point was larger than the slope up to the transition point. Boxplots of individual participants’ performance slopes before and after the transition point are shown in Fig 9. We found that for 16 of 23 participants, the slope was larger beyond the transition point than preceding it. We also assessed whether the slopes before and beyond the transition points were substantially larger than zero. We found that only 9 of 23 participants had slopes larger than 0.05 per degree of visual angle up to the transition point, whereas 19 of 23 had slopes larger than 0.05 beyond the transition point.

**Fig 10:**
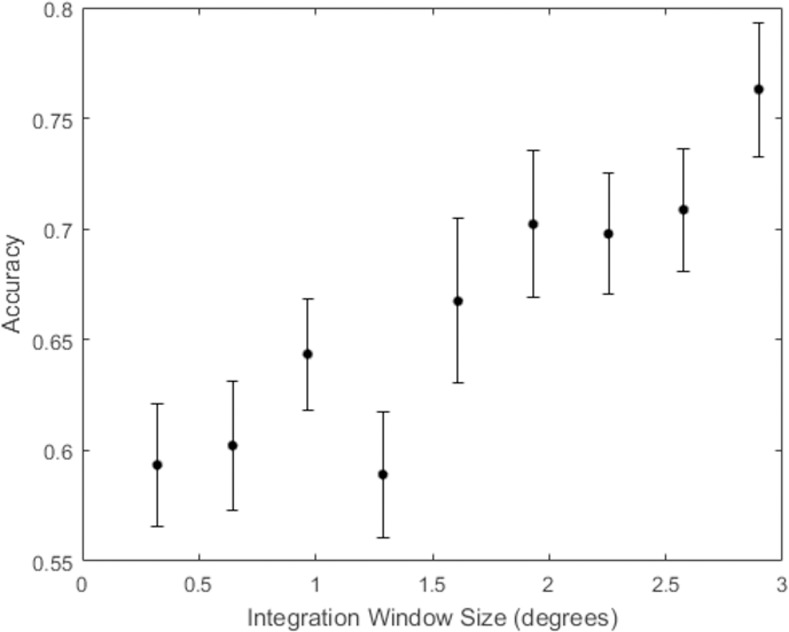
Experiment 2 results with a constant curvature target. The error bars reflect ±one standard error of the mean.

We also analyzed the data for when participants saw a shape made of constant curvature segments first and would thus be correct in choosing the CC shape representation in the 2AFC task. This condition was included primarily as a control to prevent participants from using a strategy of choosing a shape with nonconstant curvature in the 2AFC task without encoding the shape in the first display, but we were interested to see if participants confused the constant curvature target with the nonconstant curvature distractor at around the same value for *W* in these trials. The results are shown in Fig 10. Overall performance was slightly higher for constant curvature targets than for nonconstant curvature targets, although a repeated measures ANOVA did not find a reliable main effect for target type (*F*(1, 176) = 3.19, *p* = .09). Piecewise linear regression analyses confirmed that the most variance was explained when a transition point was placed at *W* = 1.29, just as in trials with a nonconstant curvature target (*R* = .126 for continuous piecewise models or *R* = .96 for noncontinuous piecewise models).

## Discussion

In Experiment 2, we sought to fix the constant curvature model’s second free parameter, the size of the integration window used to segment a contour into CC primitives. In the computational model, segmentation based on window size precedes CC segment merging governed by the threshold parameter we estimated in Experiment 1. The segmentation is an intermediate output in the model, and therefore not directly testable, so we estimated the threshold parameter first, then fixed it in the model for Experiment 2. Even though participants were tested on model outputs with both parameters, only the integration window size was varied between trials, so performance differences can only be explained by the segmentation parameter.

Participants had to encode an open contour, and then match that contour in a two alternative forced choice task. The distractor in the 2AFC task was always a constant curvature representation of the contour made with a varied integration window size and a fixed merging threshold parameter. When the integration window was small, participants’ encoded contour representation was indistinguishable from the constant curvature representation, despite substantial physical differences between the original stimulus and the constant curvature stimulus. For example, the constant curvature representation generated with a 0.97-degree integration window could not be discriminated from the original shape contour at better than chance rate, despite having, on average, 3.7% as many unique curvatures (9.83 vs. 266.45).

What integration window size is most likely to be used by the visual system for constant curvature segmentation? Any window size that falls along the positive slope region of our regression is likely too large because participants perceive a difference between the constant curvature contour and the representation they have encoded. On the other hand, if two model outputs are equally indistinguishable from the encoded contour representation, the visual system is most likely using the simpler model output for reasons of efficiency. The best choice for the integration window size, then, is the point of slope change in our continuous piecewise regression analysis, or 1.29 degrees of contour length.

Analysis of the trials with a constant curvature first display provide converging support for a transition point at 1.29 degrees. In these trials, participants compared their representation of a constant curvature contour fragment with the same contour fragment and a similar nonconstant curvature fragment. Though the task did not require participants to store the nonconstant curvature fragment in visual memory, it would still have required recoding as a set of constant curvature segments to be perceived, so participants should have had difficulty judging between the constant curvature target and the nonconstant curvature distractor for sufficiently small integration window sizes. Analyses of the data where a constant curvature contour fragment was shown first confirmed that participants’ performance began to change beyond integration window sizes of 1.29 degrees.

Overall performance was higher for constant curvature targets than for nonconstant targets, although this difference was marginal. One possibility is that encoding a constant curvature target is easier than encoding a nonconstant curvature target because it has less curvature variation. The data from Experiment 2 are not enough alone to support this hypothesis, but recent results from a visual search paradigm support this conjecture [46], and a different shape memory task designed to test this idea more directly supports the hypothesis that visual memory is better for contours made up of constant curvature constituents than contours with nonconstant curvature [45].

In Experiment 2, we operationalized the integration window size as an absolute measure of length in terms of degrees of visual angle. This is likely not how it is used in the visual system. We would not, for example, expect a difference in segmentation for a shape viewed at different distances. There is a great deal of evidence that shape representations are scale invariant [15–19]. One possibility is that the size of the integration window is a percentage of the overall contour. The 1.29 degrees in our data would correspond to 10% of the contour’s total length.

Issues arrive, however, when we use contour fragments as in this experiment. It seems unlikely that the visual system would segment a contour differently if it was fragmented from a larger contour or if part of the contour was covered by an occluder. Ideally, the integration window size would be invariant to changes in scale, while still depending only on curvatures relatively nearby to it. One way we could correct for this is by using window sizes that are a percentage of the contour but adjusted by the sum of the turning angle within that contour. Since object contours tend to be closed, we would expect turning angles along the whole contour to sum to 2π radians (360 deg). We can therefore estimate the amount of the contour visible as a ratio of the sum of the turning angle for the visible contour to 2π. In Experiment 2, for example, the average turning angle for the open contours participants were shown was 2.48 radians, or 39.5% of the whole contour’s length. Then, instead of fixing the integration window size at 10% of the visible contour, we can fix it at 3.95% of the whole contour. We use this formulation going forward because it allows us to make scale invariant segmentations that are nonetheless consistent across fragmentation and partial occlusion.

## Experiment 3

Experiments 1 and 2 aimed to fix the two free parameters in the constant curvature model. In Experiment 3, we tested whether the parameterized constant curvature model can explain important aspects of human shape perception. Previous work found converging evidence across several research paradigms for the role of constant curvature segments as primitives of shape representations [45, 46]. However, previously reported studies could not test predictions based on specific model outputs because the window size and curvature threshold were not known and therefore could not be set in the model. Using the estimated parameters from Experiments 1 and 2, we tested whether features of the constant curvature representation of a shape pair could predict human performance in a matching task.

Based on the results of Experiment 2, we used the segmentation rule in (4), where C is the total length of the shape’s contour. Following the initial segmentation, we merged adjacent constant curvature regions together if their curvatures were sufficiently similar. Using the results from Experiment 1, we used the merge rule in (5). The version of the model we tested in Experiment 3 had no other free parameters.

$$\left({\text{k}}_{\text{a}}-\frac{1}{2*\left(.04*C\right)+1}\sum_{i=a-\left(.04*C\right)}^{i=a+\left(.04*C\right)}k_{\text{i}}\right)*\left({\text{k}}_{\text{b}}-\frac{1}{2\left(.04*C\right)+1}\sum_{i=b-\left(.04*C\right)}^{i=b+\left(.04*C\right)}k_{\text{i}}\right)<0$$ (4)

$$\frac{\max\left(k1,k2\right)}{\min\left(k1,k2\right)}<1.18$$ (5)

We tested whether differences in a shape that necessitate a new constant curvature segment in our model are more detectable than shape differences over which the constant curvature model ultimately abstracts (i.e., encodes as belonging to the same constant curvature segment). We generated pairs of shapes by deforming the contour of a novel shape by a small amount. We then subjected both members of the pair to the parameterized constant curvature model to determine whether the deformation necessitated fewer or more constant curvature segments in the representation. We tested participants’ sensitivity to a difference in shape for pairs that differed by zero, one, two, three, or four segments in the constant curvature representation produced by the model. If our model and parameters are accurate, we predicted that shape pairs that differed by more constant curvature segments would be easier to discriminate from each other than shape pairs that differed by fewer segments, even if the physical difference between pairs was equated in both conditions. In generating a large number of random shapes, there will generally be a trend such that a larger difference in segment number will correspond to a larger physical difference in two shapes’ contours. To control for this association, we equated the amount of physical difference in pairs of shape contours across conditions before testing whether two shapes that differed more in segment number were more perceptually different despite being equally physically different.

## Methods

### Participants

Twenty-three undergraduates (7 male, 16 female, *M*_*age*_ = 19.65) from the University of California, Los Angeles participated in Experiment 3 for course credit. All participants had normal or corrected-to-normal vision.

### Display and apparatus

All display conditions were the same as in Experiment 1.

### Design

There were 250 trials in total. Experiment 3 consisted of six conditions which differed in the segment number difference between a pair of shapes. In the first condition (125 trials), the two shapes were identical. In the other five conditions (25 trials each), the shapes were different. Conditions were separated based on the difference in number of segments between the two shapes in a pair, from zero to four. All conditions were interleaved with each other, and participants were never informed of these different conditions. Before beginning the main experiment, participants completed five practice trials.

### Stimuli

Whole shapes were generated as in Experiment 2, by shifting 12 control points a random distance from a circle and radially fitting cubic splines between them. None of the contour regions in the shapes had constant curvature. “Different” shape pairs were generated by moving two adjacent control points a random distance to maintain equal contour length, and re-fitting a cubic spline between the new set of 12 control points.

For the different shapes, we wanted to create shape pairs that were equally different from each other across conditions. When we randomly generate shapes and subject them to the model, various displays will have different amounts of deviation in the constant curvature representation from the physically given stimulus. They also may have different numbers of constant curvature segments. We wanted to create shape pairs that had equal physical similarity, on some reasonable objective measure, despite differing in number of segments. In other words, if in the condition where one shape had one more constant curvature segment than its paired shape, and if the two members of the pair were 95% similar according to an objective measure of physical difference, then pairs of shapes in conditions in which the segment number difference was zero, two, three, or four should also have an average similarity of 95%. To this end, we imposed a constraint on the physical difference between shape pairs, discarding pairs that were too physically different. We computed physical contour similarity by taking the ratio of the overlapping areas to the non-overlapping areas for both contours. Since this measure is asymmetrical, we computed the average:

$$\frac{\frac{\text{Shape}\;1\;\text{and}\;2\;\text{overlap}}{\text{Total}\;\text{area}\;\text{of}\;\text{Shape}\;1}+\frac{\text{Shape}\;1\;\text{and}\;2\;\text{overlap}}{\text{Total}\;\text{area}\;\text{of}\;\text{Shape}\;2}}{2}.$$

**Fig 11:**
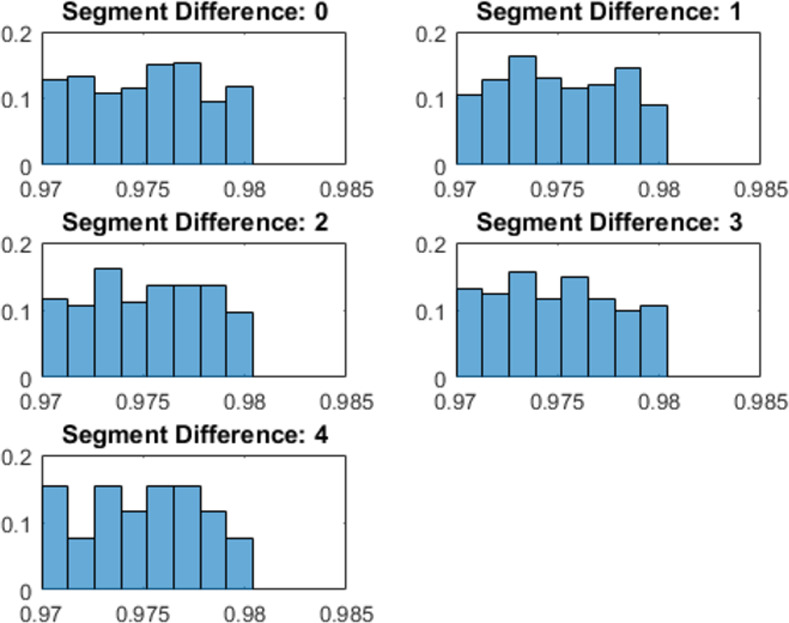
Histogram of physical similarity between pairs of shapes for each experimental condition in Experiment 3. Each histogram shows the distribution of similarities between shape pairs for each of the five experimental conditions. The x-axis gives the amount of physical contour difference between members of a pair, and the y-axis for each histogram gives the proportion of shape pairs with that amount of difference.

**Fig 12:**
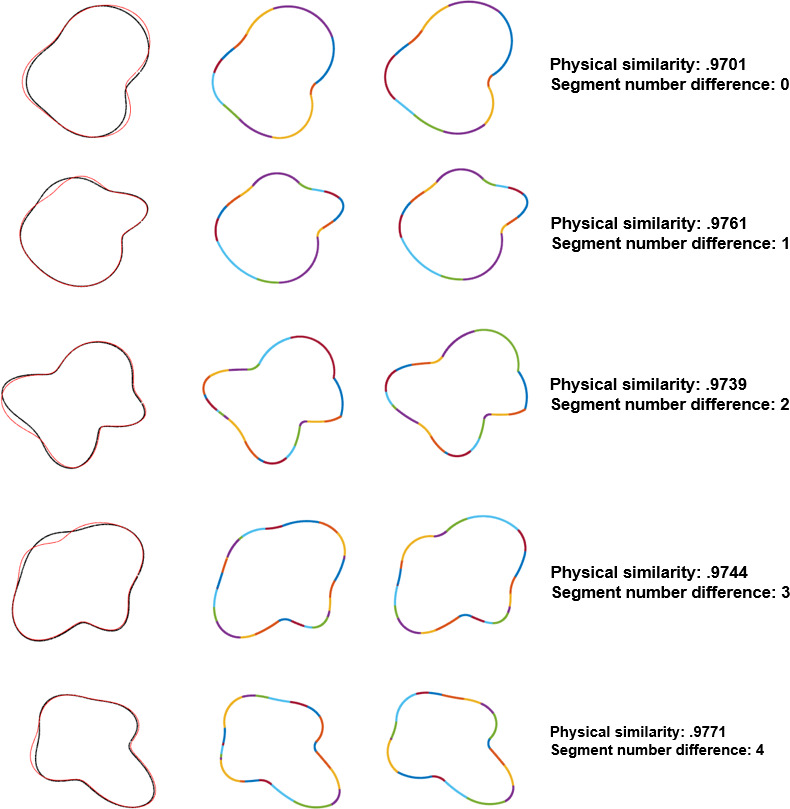
Sample shape pairs from the five experimental conditions in Experiment 3. In the left column, we show the two shapes overlaid on top of each other (Shape 1 in black, Shape 2 in red) to show the physical differences between the two contours. In the middle column, we show the constant curvature representation of Shape 1. In the right column, we show the constant curvature representation of Shape 2. *Physical similarity* refers to the physical contour difference between the two shapes, as measured by the overlap of their interiors. *Segment number difference* refers to the difference in the number of constant curvature segments between the two shapes using the parameterized constant curvature model.

We generated hundreds of shape pairs and categorized them based on their constant curvature segment difference. We computed the segment difference by using the threshold parameter of 1.18:1 from Experiment 1 and the integration window size parameter of 4% of the whole contour from Experiment 2. After sorting them into five categories corresponding to a difference in number of segments of zero, one, two, three, or four, we confirmed that the physical difference of shape pairs was matched across categories. Mean difference and standard deviation for all five categories were matched to a hundredth of a percent at 97.51% similarity and 0.29% deviation. Fig 11 shows a histogram of the similarity between pairs in each of the five experimental conditions. Sample pairs from each condition are shown in Fig 12.

In structural models of shape, there are two possible kinds of shape differences. A qualitative shape difference can be thought of as a change in the number of shape primitives composing the object, such as the addition or deletion of a constant curvature segment in our model, or of an axial branch in skeletal shape models. A metric shape difference is a change in the features of a shape primitive (see [31] for discussion). In our model, this might correspond to changes in the curvature or angular extent of one of the segments. The same amount of physical shape change can be achieved by different amounts of qualitative and metric shape changes. We expected that even though these changes give rise to the same amount of contour differences, qualitative changes (i.e., changes in the number of segments in a representation) will produce more perceptually different shape representations than metric changes.

### Procedure

**Fig 13:**
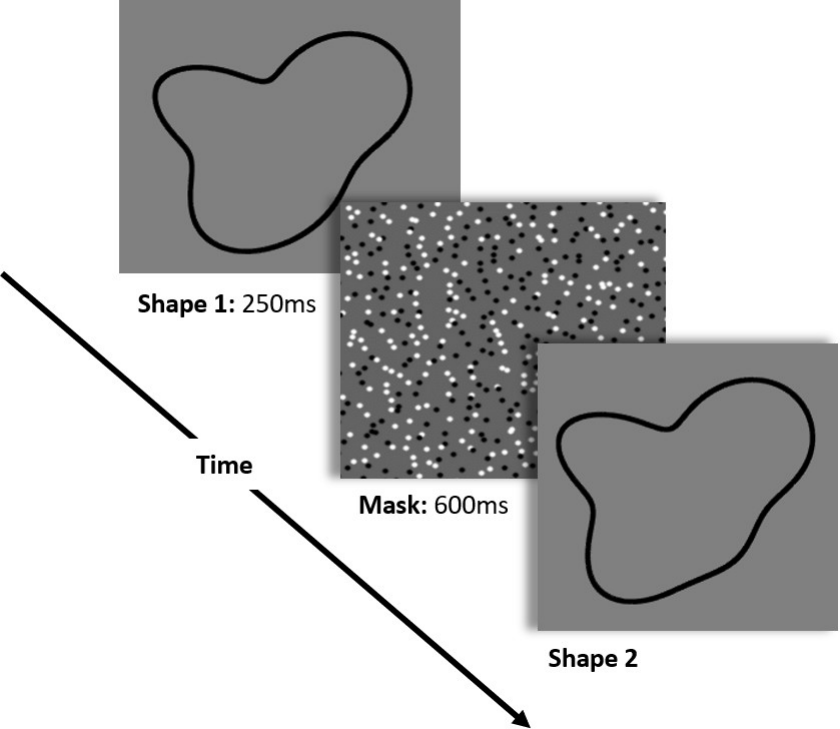
Sample trial from Experiment 3. Participants were shown the first member of the shape pair briefly, for 250 ms, followed by a pattern mask. Participants were then showed the second member of the shape pair, which remained on the screen until they responded. Participants were asked to report whether the second shape was the same or different from the first.

On different trials, participants were shown sequentially both members of a pair of shapes and were asked to judge if they were the same or different. Though equally physically similar, a pair of shapes could have a segment number difference between zero and four. On ‘same’ trials, participants were shown exactly the same shape twice. Each trial began with a fixation cross shown in the center of the screen for 500 ms, followed by the first shape, which remained on the screen for 250 ms. The first shape was then masked by a pattern of black and white dots (600 ms), after which the second shape was displayed, along with a prompt at the top of the screen asking participants to decide if the second shape was the same or different as the first shape they had been shown. Participants were instructed to press A if the two shapes were the same, or L if the shapes were different. The second shape remained on the screen until participants responded. Fig 13 shows a sample trial. Participants completed five practice trials with the experimenter present to make sure all instructions were understood before beginning the main experiment.

## Results

**Fig 14:**
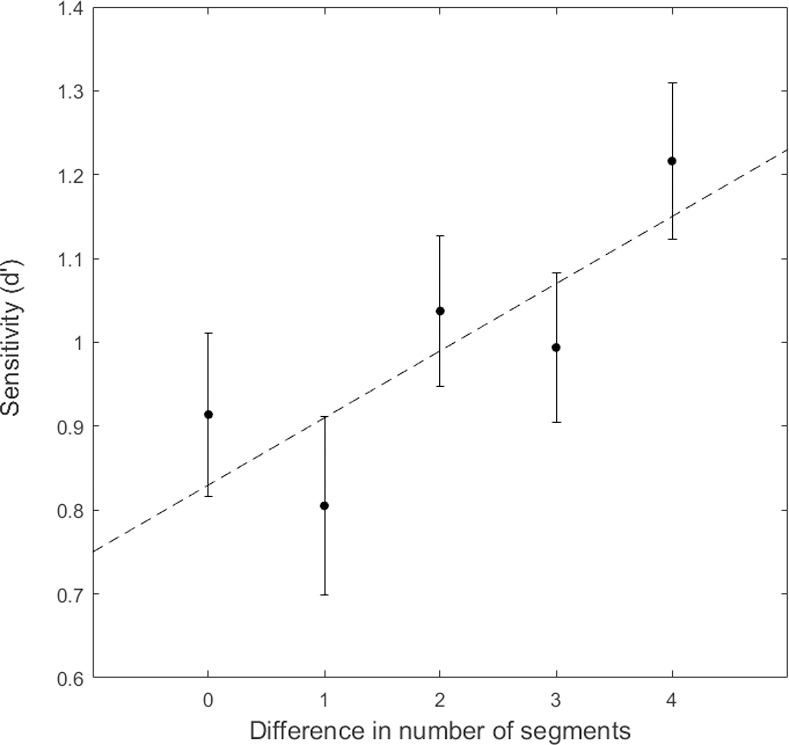
Results of Experiment 3. d’ is plotted as a function of the difference in number of constant curvature segments between a pair of shapes. Error bars show ± 1 standard error of the mean. The dashed line shows a linear fit to the data.

One participant’s data was not analyzed because she was at chance performance across all conditions. The overall results are not affected by her inclusion or exclusion. We analyzed the data from Experiment 3 by computing participants’ sensitivity to a shape change between the first and second display. A hit was classified as a correct “different” response when the shape had changed, and a false alarm was classified as an incorrect “different” response when the second shape was the same as the first. We computed d’ for each condition as Z(hit)–Z(false alarm). The results are shown in Fig 14.

**Fig 15:**
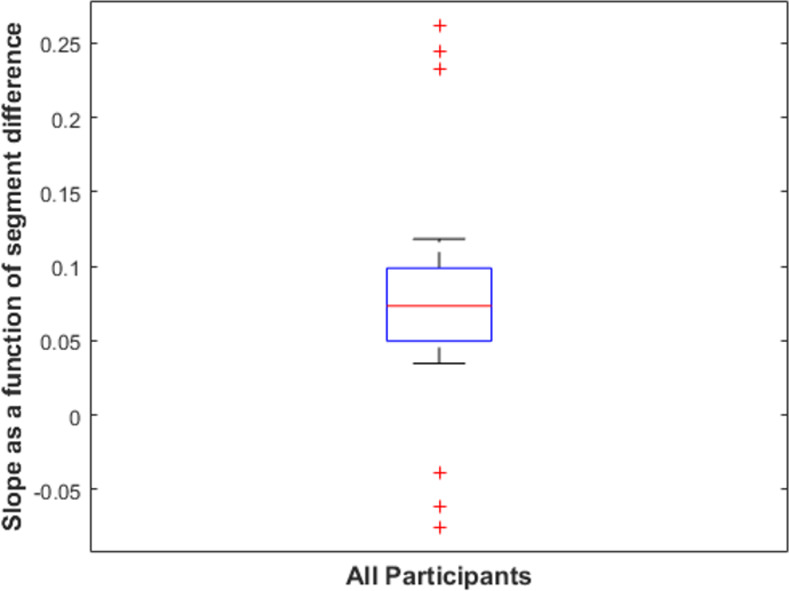
Boxplot of participants’ performance slopes as a function of constant curvature segment difference. The box shows the interquartile range of slopes for individual participants. The red line shows the sample median. The red line shows the sample median. The whiskers extend to the most extreme datapoint within 1.5 times the length of the interquartile range from the top or bottom edge of the box (covering 99.3% of the data if they are normally distributed). Outliers are data points beyond the whisker and are plotted as red +’s.

To analyze whether there was a significant effect of segment number difference on participants’ ability to detect a shape change, we fit a linear regression function to our data. The data were best fit by the function y = 0.83 + 0.08x. A repeated measures ANOVA confirmed a significant linear component, *F(1*,*20)* = 17.62, *p* < .001, η^2^_partial_ **=** .468, indicating a significant positive change in performance as the difference in segments gets larger. To confirm that the positive slope was not a result of a few anomalous datapoints, we analyzed the distribution of slopes across individual participants. A boxplot of participants’ slopes as a function of segment differences is shown in Fig 15. As the figure shows, all but three statistical outliers have a positive slope, consistent with better performance for more representationally different shape pairs. We also conducted a nonparametric chi-square analysis to test whether more positive slopes were observed in the data than would be expected if there was no correlation between performance and segment number difference. We found that there were significantly more positive slopes in the data from Experiment 3 than would be expected without a correlation, χ^2^(1, N = 23) = 12.57, *p* < .001.

**Fig 16:**
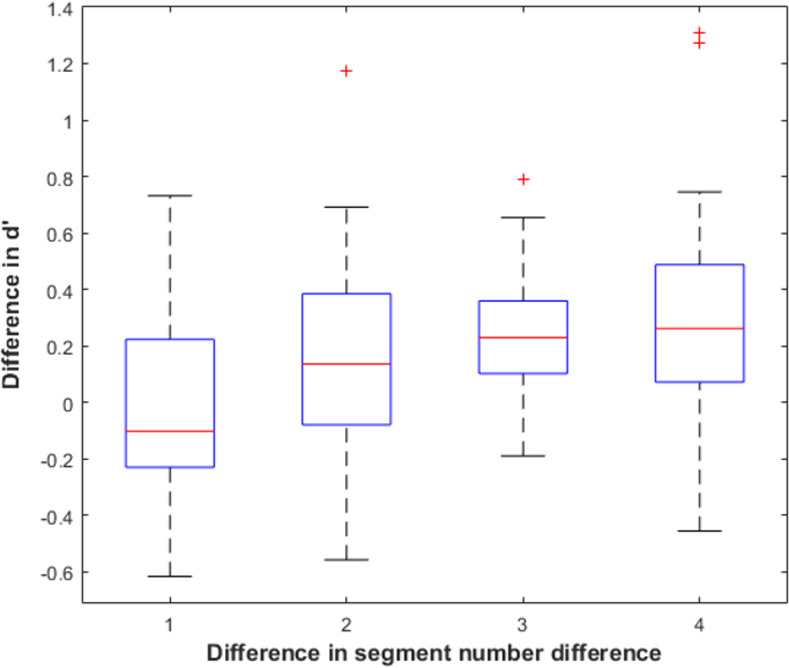
Sensitivity difference by segment number difference. The box shows the interquartile range of d’ differences for individual participants. The red line shows the sample median. The whiskers extend to the most extreme datapoint within 1.5 times the length of the interquartile range from the top or bottom edge of the box (covering 99.3% of the data if they are normally distributed). Outliers are data points beyond the whisker and are plotted as red +’s.

We also directly compared particcipants’ sensitivity for pairs with differing amounts of segment number differences. We found the average difference in sensitivity between trials of a certain segment number difference and trials in which the segment number difference was one, two, three, or four segments higher. For example, for a difference in segment number of one, we averaged across comparisons of 0 vs. 1 segments, 1 vs. 2 segments, 2 vs. 3 segments, etc., whereas for a segment difference of two, we averaged across comparisons of zero vs. 2 segments, 1 vs. 3, segments, and 2 vs. 4 segments. The motivation for this analysis was that we did not know *a priori* what amount of difference between numbers of segments would be salient. As Fig 14 suggests, a difference in paired shapes of only one segment may have a relatively subtle effect. If segment number in our model does relate to representational differences, however, we expected clearer differentiation of pairs as their segment differences increased. Results of these comparisons are displayed in Fig 16. A one-way repeated measures ANOVA confirmed a significant main effect for difference in segment number differences such that mean sensitivity was higher when shapes differed by more constant curvature segments, F(3, 60) = 4.25, p = .009, η^2^_partial_ = .175. There was also both a significant linear and quadratic relationship between segment number differences and d’, F(1, 20) = 5.57, p = .029, η^2^_partial_ = .218 for linear and F(1, 20) = 8.05, p = .01, η^2^_partial_ = .29 for quadratic.

## Discussion

Experiments 1 and 2 were designed to estimate experimentally the two free parameters in our computational model of constant curvature encoding of contour shape. In Experiment 3, we set the model’s parameters to fixed values based on the estimates from the earlier experiments, and we aimed to assess the resulting model by testing whether its outputs could explain aspects of human perceptual capabilities.

In our model, some differences in curvature are abstracted over as shapes are recoded into constant curvature regions, while other curvature differences necessitate a segment boundary. We predicted that those differences that result in a change to the number of constant curvature segments should be more salient to viewers. Consequently, when we generated shape pairs by deforming a contour, we predicted that if the contour changed did not lead to a segment number difference in the two shapes’ constant curvature representation, a difference between the two shapes should be harder to detect than in a pair of shapes where the deformation resulted in the gain or loss of constant curvature segments. Moreover, we predicted that larger changes in segment numbers should make shape differences more detectable.

As a control, we equated the amount of physical dissimilarity between shapes in a pair across the five experimental conditions. The members of the pair were highly similar, with an average of 97% physical contour similarity. We chose to use highly similar shape pairs in order to make the shape matching task difficult enough for differences across the experimental conditions to emerge. While larger shape differences may have made the task too easy for participants, the constant curvature model is well suited to capture such differences: Shapes that are more physically dissimilar also have more dissimilar constant curvature representations. As confirmation of this, we generated a pair of shapes with 71.5% contour similarity and found that they differed by six constant curvature segments and the curvature and extent of individual segments differed by considerably more than for pairs of shapes that were 97% similar.

Participants made same/different judgments for sequentially shown shape pairs. Participants were never told anything about the constant curvature model or given any kind of indication that the “different” shape pairs fell into five distinct categories. Nevertheless, they showed reliable differences in performance as the difference in numbers of constant curvature segments increased. Importantly, differences in constant curvature number did not correlate with the magnitude of physical contour differences in this experiment. The distribution of contour differences was identical across all five conditions. Performance differences must therefore be explained by how shapes are perceived and encoded, not by a generic measure of overall physical differences in the contour.

Although the number of constant curvature segments generated in the model reliably predicted discriminability, the increase in performance was gradual, as shown by the modest slope of the regression line in Fig 10. In fact, the data are consistent with the idea that a representational difference of a single segment was not a strong driver of discrimination performance. This was confirmed in an additional analysis, in which all such differences (of a single segment) were compared with differences of 2, 3, and 4 segments. This analysis revealed that across the experiment, differences of a single segment did not reliably produce performance differences, but increasing differences in the number of segments produced monotonically increasing performance differences. It is important to recognize that the construct of *n* segment differences between two shapes is entirely derived from the specific model we use here. These numbers of segment differences depend both on the concept of encoding contour shapes into constant curvature segments and on the specific parameters we applied in this model. Although it is perhaps imaginable that the predictions of our model are related to some contour features that could be picked up by some other conceptual model, we know of no other such model, and it is not at all clear how or why some other approach would correlate, unless it involved the coding of contour shape into constant curvature segments.

The results of Experiment 3 depended intrinsically on the parameters we fixed from the psychophysical results obtained in Experiments 1 and 2. Both the segmentation window and curvature threshold parameter balance representational efficiency with representational accuracy. In Experiment 3, if the outputted representations were too simple, contour differences that were important to observers’ shape representations would have been captured and shape pairs that were perceptually different would often have had the same number of curvature segments. If the representations were too precise, the model outputs would not have sufficiently diverged from physical contour differences, making it difficult to predict or detect performance differences between shape pairs whose physical contour similarities are equated.

## General discussion

The present work was motivated by emerging evidence that visually perceived contour shape is represented by constant curvature segments [45, 46]. This idea is also consistent with neurophysiological findings [49], ecological considerations [47, 50, 51] and other psychophysical research [66, 67]. In the current work, we explored further whether constant curvature segments were plausible primitives for shape representation by developing and assessing a specific computational model of shape encoding consistent with human performance.

A model that represents shape contours in general as being made up of constant curvature segments would obviously be well-suited for viewed contours that actually *are* comprised of constant curvature parts. In general, however, contours in viewed scenes are not of constant curvature; most often they have continuously changing curvature. Much of the work, then, of representing shape contours via constant curvature segments consists in “falsifying the world” in the sense of encoding contours not comprised of constant curvature segments in terms of a representation that consists of such segments. The benefits of coding in this way include obtaining an economical structural description that can nonetheless be a reasonable approximation to the stimulus. Inevitably, there is a tradeoff between having a very large number of short constant curvature segments, which, in the limit, would very closely capture the physical input, vs. having a smaller number that provides a more compact description, yet still captures shape with some degree of fidelity.

Two parameters in the model govern the precision of segmentation and grouping of curvature. One of these parameters, *W*, indicates the extent along the contour that is considered in determining the locations of contour inflection points. The other parameter, *T*, operates within singly inflected segments yielded using *W*. *T* reflects the threshold for how different adjacent parts within a constantly inflected segment need to be in order to be represented as different constant curvature segments.

Both of these parameters influence the precision of segmentation. Smaller values of *W* and *T* lead to more precise representations, having higher fidelity to the physical stimulus, but at the cost of more detailed representations, i.e., more numerous and smaller constant curvature segments. Prior to the present work, we thought it likely that no single assignment of parameters would likely be feasible in the model, as the precision of shape representation itself might vary with task, focal attention, learning, individual observers, interactions of these, or other factors. Although task variation may yet prove important in certain contexts, here we explored the possibility that, within some range of paradigms assessing ordinary shape discrimination and shape comparisons, there might be consistent values of these parameters. The results of the experiments supported this idea. Across the range of tasks used in the experiments here, there was substantial consistency across naïve observers in both tasks, allowing us to obtain psychophysical estimates of the parameters *W* and *T*.

Experiment 1 allowed us to estimate the value of T, curvature variation below which contour segments are represented by perceivers as having a single curvature. The experiment asked observers to choose which of two contours appeared more complex. Stimuli always consisted of one segment of constant curvature and another made from two joined segments of differing curvature. We reasoned that curvature differences that were noticeable would likely drive these judgments, and that below some level of curvature variation, complexity judgments would go to chance level between single curvature contours and those made of two curvature segments. Despite our giving them no instructions on how to interpret “complexity,” participants were consistent and systematic in showing responses based on curvature differences. Using the 75% threshold, we set parameter *T* at a 1.18:1 ratio of curvatures in the model.

The subjective notion of contour complexity used in our Exp. 1 differs from previous theories in which complexity depends on the amount of curvature within a contour [27, 28, 68]. Under this view, whether the two-segment contour or the one-segment contour was more complex should depend only on whether the curvature in the one-segment contour was higher than the mean of the curvatures in the two-segment contours. In Feldman and Singh’s derivation, a straight-line continuation of the contour in the direction tangent to the last point adds the least new information [28]. Within the context of our experimental paradigm, participants’ subjective reports suggested that a continuation in the last point’s curvature added the least amount of new information.

Experiment 2 was constructed to allow us to estimate *W*, the integration window for initial segmentation based on contour inflection. In the model, *W* operates on 2D contours and divides them, roughly speaking, into segments of similar curvature values. If the window is small, the model has low tolerance for curvature variation and divides the contour into many small pieces. As the window gets larger, it abstracts over more curvature variation, dividing the contour into fewer pieces. We estimated the integration window size that most closely corresponds to shape encoding in human perception by comparing participants’ ability to discriminate between a contour with no constant curvature and a constant curvature representation of the contour produced from various window sizes. We hypothesized that the visual system uses the largest window size at which the constant curvature representation is not distinguishable from the original contour, so we fixed *W* at the transition point between the region of the psychometric function that showed near-chance responding and near-zero slope and the region in which discrimination performance increased monotonically with window size.

A side benefit of Exp. 2 is that participants’ difficulty in distinguishing between contour fragments and their constant curvature representations under some values of *W* furnishes additional evidence that constant curvature primitives are used in human shape encoding. Other primitives like straight lines (e.g., [42, 43]) could also approximate the contour well enough to be indistinguishable from the original, but likely with far more components. Constant curvature representations of 2D contour shapes that could not be discriminated from the original contour were still relatively economical in our results, generally consisting of eight to 12 segments.

Having fixed the two parameters of our model based on psychophysical data, we tested the full model in Experiment 3. To do this, we tested participants’ performance in a speeded same/different task. We generated shape pairs by randomly creating a first shape and then applying a random distortion to it to produce another shape that had the same contour length and was 97.5% similar in terms of shape overlap. For many such pairs, we computed the number of CC segments in each member of the pair. We then sorted the pairs into five categories based on the difference in segments between the shapes in the pair. Importantly, shapes in all five categories were equated in terms of physical contour difference; the amount of contour overlap between pairs that had zero difference in numbers of segments was identical to that of pairs that differed by 5 segments.

Because pairs were equated in this way, shape coding schemes other than what we have proposed would be expected to predict no differences in same/different classification for the various pairs in the experiment. Our independent variable relating to differing numbers of constant curvature segments in a representation exists only within our theoretical framework. If, however, shape representations utilize such a representational scheme, we predicted that participants should be better at detecting differences between shape pairs that had larger differences in the number of segments in their CC descriptions. The results of Experiment 3 supported this prediction, showing a reliable linear trend in which shape pairs with smaller differences in CC segment number were more difficult to distinguish than shape pairs with larger segment number differences. The model was able to identify contour differences that were perceptually salient, even when those differences do not correspond to larger changes to the physical contour. An additional analysis showed that, whereas the performance differences for pairs differing by only one segment in the theorized representation were small, performance was clearly and monotonically enhanced by differences of 2, 3, or 4 segments in the representation produced by the model.

Taken together, these results indicate the plausibility of encoding arbitrary 2D contours and closed 2D shapes as sets of constant curvature segments. The computational model put forth here not only produces these descriptions but does so based on parameters that agree with psychophysical data about shape discrimination. The resulting model was able to predict performance in a new shape discrimination task based on numbers of components in the theorized representations of 2D shapes.

### Limitations

Although the findings of our experiments provide evidence for constant curvature shape processing and the model described here, we must note that this model does not exclude other approaches to shape, such as skeletal or structural models. The constant curvature model aims to provide an account of the initial abstract representation of contour shape. Such a representation likely provides the input for other shape computations, as in detection of symmetry, or, more broadly, in the kinds of coding described by structural information theory [69, 70]. For some kinds of shapes and transformations, further processing might be needed to provide invariance under articulation, a task to which skeletal models are well-suited, or to nonrigid transformations of the object, a task that might involve some inference of the shape’s history [71], or to allow recognition for objects in the same basic category [72], a task better suited to structural descriptions (e.g., [73]). We hypothesize that all further shape processing builds upon the initial abstract description of contour shape described here, rather than proceeding directly from the earliest, local, subsymbolic activations (see below) produced by the stimulus.

The constant curvature model described in this study also does not explain how shape descriptions are obtained from contours with many local contour features. For example, adding small serrations or sinusoidal modulations with low amplitude and high frequency would be unlikely to affect viewers’ representation of an object’s global shape [74], but it would significantly change the constant curvature description of the contour put forward by our model. This issue is of particular importance because natural objects typically have a great deal of local features along their bounding contours, such as fur on a dog, twigs on a tree, or wisps on a cloud. Recent research has found some evidence that local and global contour information is processed independently and by separate systems in visual perception [74, 75]. The constant curvature shape description put forward would operate in the global processing system, not the local one. In the current study, we used contours in which such local contour perturbations were absent, in order to focus on other issues.

The important problem of how the visual system abstracts away local contour features is a question we are currently working towards answering. One possibility is that local contour features are described as a statistical distribution rather than individually, and the visual system ignores contour features likely to have been sampled from the local feature distribution [75]. Another, more biologically inspired possibility is that the visual system uses oriented luminance contrast detectors at multiple scales when forming a subsymbolic description of an object’s contour and discounts local contour features that are present at small scales but are not captured by larger-scale detectors.

Another limitation that is specific to the present work concerns the integration window represented by the parameter *W*. In initial efforts, we used length along a contour to define this parameter. This had undesirable effects of changes with scale. For two figures having the same shape but differing sizes, a window based on absolute contour length (e.g., in terms of visual angle) would have the unintuitive effect of utilizing a smaller part of a larger figure than it would for the smaller figure. A tentative solution, which we used in the model given here, is to specify the window size as a percentage of a closed shape contour. For open contours like the stimuli used in Experiment 2, we computed window size as a percentage of the contour fragment, then computed the percentage of the whole shape contained in the fragment. Since closed contours always have a sum turning angle of 360 degrees, we could estimate the percentage contained in a fragment by computing the sum turning angle of the open contour divided by 360 degrees. Using this correction, we found an object-centric integration window size from the data in Experiment 2, about 4% of a closed shape’s contour.

This approach worked well enough in the current model, but it is unlikely to be the correct one ultimately. We believe that a solution for finding a scale-invariant window size may be forthcoming from efforts to implement our model using as its inputs the outputs of biologically plausible neural units sensitive to oriented contrast at various locations along a contour. Classic work in neurophysiology has found that the visual system is specially tuned to straight, oriented luminance contrasts in various positions in visual field [76]. Curvature information may be in the turning angle between two contrast detectors positioned end-to-end along a contour [66]. Crucially, oriented edged detectors operate across different scales (e.g., [77, 78]). Different shapes might be best captured by edge detectors of different scales. For example, a shape with very high curvature likely needs small detectors to capture the rapid change along the contour, while a shape with low curvature might be captured almost equally well by larger detectors.

If we hypothesize that the visual system utilizes as one description the largest scale detectors that adequately capture the contour’s behavior, scale invariance may naturally fall out of the constant curvature model: two shapes that differ only in scale have the same number of detectors and the same turning angle between detectors, differing only in the size of the detectors [46]. In this construction, a constant curvature primitive is encoded not by its curvature and arclength, but by its turning angle and the number of detectors in the segment. Returning to the issue of integration window size, the window size might be specified neither by absolute distance, nor a percentage of the closed contour, but as a fixed number of detectors to be considered. In small shapes, the curvature is higher and the detectors will be smaller, while in large shapes the curvature will be lower and the detectors will be larger. Both shapes, however, will have the same number of detectors, and if the integration window is based on detectors considered, the segmentation will be the same.

These considerations relating to the integration window relate to a broader limitation of the present work. The model we have tested here takes as inputs mathematical curvature values at various points along the input contours. In biological vision systems, initial encodings that involve contour orientation appear first in area V1 of visual cortex. Thus, a more biologically plausible model would take the outputs of these early filters as the inputs.

To arrive at an abstract representation of contour curvature, operations on the outputs of neural units with local receptive fields sensitive to oriented contrast must somehow allow extraction of more abstract, symbolic representations of shape. This transformation has been described as involving a connection between initial subsymbolic responses (e.g., detection of oriented contrast) into symbolic representations, such as contour tokens having shapes [12]. The activations of early cortical units increase monotonically with contrast energy in a visual scene and can be thought of as direct responses to patterns of light striking the retina. While rich in information, these representations fluctuate with changes in viewing position and illumination, and they are transient, easily destroyed by masking or the passage of time [24, 79, 80]. Symbolic representations, in contrast, designate properties of material objects in the world, such as contours, shapes, and surfaces [12].

The relation between early, subsymbolic encoding of energy properties and more abstract, durable representations of properties of material objects has been mysterious in vision science, and most work proceeds on one side or the other of this divide. Contour shape encoding via constant curvature segments may offer an existence proof of how more abstract representations may arise from early, local encodings. In the current model, we have shown how such contour tokens may be obtained from a set of curvatures along the contour to a much smaller set of constant curvature regions. In other work, we have described a framework for obtaining these symbolic descriptions from initial inputs that consist only of local, orientation-sensitive neural units and their spatial relations [46]. Based on the evidence presented in this paper, and the specification of a computational model based on psychophysical results in shape discrimination, a goal of ongoing work is to develop a detailed model that mirrors the operations of the computational model given here but does so based on initial activations of neutrally plausible units. Such an effort seems valuable both in terms of understanding the important domain of contour shape perception but also in understanding how abstract, symbolic representations, such as shape, may be derived from information captured in early visual coding.
